# The ongoing journey in targeting hemodynamic interventions: missing miles for missing the last micron?

**DOI:** 10.1186/s40635-024-00621-y

**Published:** 2024-04-09

**Authors:** Johannes Heymer, Daniel Raepple

**Affiliations:** Internistische Intensivmedizin, Zentrum Für Innere Medizin, Klinikum Stuttgart, Kriegsbergstraße 60, 70174 Stuttgart, Germany

With great interest we followed the original work by Bruno et al. [1] assessing sublingual microcirculatory perfusion variables in shock and the following discussion by Hilty et al. [2]. The authors critique the study for its use of unvalidated software and the absence of an effective, hemodynamic monitoring-based treatment plan. They also highlight the study's failure to address different shock types and their unique microcirculatory characteristics, crucial for appropriate treatment decisions.

In the last decades, therapy guided by either macrocirculatory or microcirculatory targets in patients with shock has not yet shown satisfactory results to demonstrate benefits in directing treatment for these patients.

In our response, we aim to explore an alternative perspective by examining acid–base homeostasis to evaluate shock progression or resuscitation success. We propose to focus on the dynamics of Base Excess (BE) as an indicator of successful resuscitation in shock scenarios. BE is a calculated parameter that determines the amount of acid or base required to restore the blood's pH to 7.40 under standardized conditions, assuming normal CO_2_ levels. A negative BE, coupled with acidemia, indicates that the primary cause of the blood's acidity is metabolic in nature. This could be due to deranged physiological buffer systems, such as loss of bicarbonate through the gastrointestinal tract, reduced bicarbonate synthesis in the case of acute kidney injury (AKI) secondary to shock, or an increase in the strong ion difference, such as that seen in hyperchloremic acidosis resulting from resuscitation with isotonic saline. Although these elements can lead to acid–base disturbances during shock, we contend that a more crucial contributing factor is the accumulation of protons arising from impaired adenosine triphosphate (ATP) regeneration.

The daily turnover of ATP in the human body is remarkably high, underscoring ATP's crucial role as the primary energy carrier in biological processes. Typically, the human body contains about 0.1 mol of ATPa at any given moment, which is continuously cycled. The human body synthesizes and degrades an amount of ATP approximately equal to its own weight each day. We propose that the pronounced acidosis following cardiac arrest is a manifestation of this abrupt metabolic shift, in contrast to the effects or confounders mentioned above, which take some time to manifest.

Traditionally, shock has been characterized by a discrepancy between oxygen delivery (DO_2_) and consumption (VO_2_). However, we suggest to see DO_2_/VO_2_ as a partial aspect of some forms of shock but broadening this definition to encompass shock more generally as an imbalance between ATP consumption and regeneration, which results in proton accumulation during acute imbalances. This viewpoint is supported by studies showing that not all shock states display a VO_2_/DO_2_ mismatch [3]; yet often, non-survivors of shock are unable to increase VO_2_ [4]. Therefore, we want to point to the last micron of the journey from macrocirculation to oxidative phosphorylation: the mitochondria.

For the complete oxidation of glucose in the presence of oxygen, the process involves several stages: glycolysis, the Krebs (or citric acid) cycle, and oxidative phosphorylation. The simplified overall chemical equation for the complete oxidation of glucose is:

C_6_H_12_O_6_ + 6O_2_ 6 CO_2_ + 6 H_2_O + Energy (stored in ATP)

The number of ATP molecules generated from one molecule of glucose can vary but is commonly cited as approximately 36 ATP molecules. When ATP undergoes hydrolysis to form ADP and inorganic phosphate (Pi), transferring the energy from the phosphate bond to its substrate, there is a net release of one proton (H^+^) to maintain charge balance [5]. The stoichiometry of complete oxidative phosphorylation is balanced only when the protons released during ATP hydrolysis are reintroduced and regenerated in the ongoing process [6].

Lactate levels and the clearance of lactate have been the focus of extensive research, particularly in the context of shock. We also wish to acknowledge the significant contributions to the field of lactate studies. However, we argue that acidemia and hyperlactatemia, while often occurring together, are not synonymous. We suggest that, although both values are influenced by various factors, BE serves as a more accurate indicator of metabolic disturbances in shock.

In glycolysis, direct ATP synthesis happens via substrate-level phosphorylation, not oxidative phosphorylation. Here, a high-energy phosphate from a substrate molecule is directly transferred to ADP to form ATP. The conversion of pyruvate to lactate is accompanied by the reduction of NAD^+^ to NADH. The reduction of pyruvate into lactate is a process that consumes protons. When pyruvate is reduced to lactate by lactate dehydrogenase, NADH donates electrons and is oxidized back to NAD^+^, and a proton (H^+^) is used up. Thus, the term “lactic acidosis” [7, 8] can be misleading: lactate production only leads to proton accumulation if the protons released during the subsequent ATP hydrolysis are not regenerated in complete oxidative phosphorylation (under conditions of oxygen shortage or dysfunctional mitochondria) or in gluconeogenesis [5, 6, 9].

If we dissect now the processes from macrocirculation to the generation of the primary energy currency, ATP, through glycolysis or oxidative phosphorylation, we observe a multi-step cascade that involves various physiological processes.

Cardiac power output (CPO) signifies the heart's capacity to perform work and serves as an emblematic measure of the "energy" present within the macrocirculatory system. It is derived from the product of the cardiac output (CO) and the perfusion pressure (P_perf_). Notably, P_perf_ is calculated as the difference between the mean arterial pressure (MAP) and the right atrial pressure (RAP). Therefore, the equation for CPO is CPO = CO × (MAP − RAP) [10]. P_perf_, in essence, is the effective pressure pivotal in surpassing the intrinsic auto-regulatory thresholds of organs and tissues.

When CPO is reduced due to a decrease in CO—stemming from conditions such as ischemic pump failure, septic cardiomyopathy, inadequate left ventricular filling, or intrathoracic obstructions—the body may attempt to maintain P_perf_. The primary strategy for increasing P_perf_ involves raising systemic vascular resistance (SVR). Clinically, this compensation is evident as "centralization," a state often indicated by cold extremities, delayed capillary refill, and skin mottling in patients. This can create a misleading impression where P_perf_ meets or exceeds conventional targets, presenting a facade of stability: when CPO is constrained and P_perf_ is artificially maintained, actual blood flow is likely compromised. Oxygen delivery (DO_2_), a subsequent factor in microcirculation, considers that blood carrying a specific amount of oxygen (C_a_O_2_) at a given flow rate (CO) is responsible for oxygen transport to organs and tissues.

Although measurement of macrocirulatory parameters, capillary flow parameters or arterio-venous CO_2_ difference [11] may act as indicators for reduced blood or oxygen transport to organs and tissues, these do not fully reflect the efficiency of the subsequent processes necessary for adequate ATP formation from substrates: namely, the mitochondria.

Mitochondrial function may be compromised by either their reduced numbers, resulting from limited microcirculatory flow, or by the availability of substrates (such as O_2_ or Krebs cycle substrates) or their impaired functionality—in the context of the latter, we want to acknowledge the concept of cytopathic dysoxia, as introduced by Fink [12]. During shock or ischemia–reperfusion injuries in resuscitation efforts, numerous elements may contribute to an inherent defect in cellular and mitochondrial respiration. These factors include nitric oxide, pro-inflammatory cytokines such as tumor necrosis factor (TNF), interleukin-1 beta (IL-1β), and interferon-gamma (IFN-γ), as well as endotoxins and intracellular acidosis, which are known to affect mitochondrial activity, as observed in conditions like septic cardiomyopathy [13].

Given these insights, we advocate for a deeper examination BE dynamics, which can be readily assessed through routine blood gas analysis, as a crucial surrogate marker for guiding future resuscitation strategies. Our view is that targeting BE could be effective in enhancing CO, provided perfusion pressure P_perf_ is maintained at levels sufficient to surpass the autoregulatory thresholds of vital organs and to optimize microcirculatory perfusion by minimizing vasoconstriction. We specifically emphasize that attempting to normalize BE solely through buffering methods, such as using sodium bicarbonate, fails to address the underlying cause and might not only be ineffective but also potentially harmful [14].

We suggest pursuing further research into this approach in patients undergoing extracorporeal resuscitation (eCPR). Our hypothesis is that in these patients, aiming to normalize BE by targeting the highest possible extracorporeal life support (ECLS) flow rates, while minimizing the use of vasopressors to preserve microcirculation, could provide valuable insights.

## Data Availability

The manuscript is designed for the series titled "Tissue Oxygenation: How to Measure, How Much to Target" and is conceptualized as a hypothesis. Consequently, it does not include sections on data or materials.
